# Assessing the spiritual and mental health of the KwaZulu-Natal flood disaster survivors

**DOI:** 10.4102/jamba.v15i1.1435

**Published:** 2023-12-18

**Authors:** Annelene van Straten, Alice Ncube

**Affiliations:** 1 Disaster Management and Training Centre for Africa, Faculty of Natural and Agricultural Science, University of the Free State, Bloemfontein, South Africa

The spiritual and mental health of flood disaster survivors has become a prominent topic among researchers as the impacts reach beyond that of economics. The research focused on the 2022 flood disaster that occurred in KwaZulu-Natal, South Africa. The disaster was declared a provincial and later a national disaster. The floods resulted in high fatalities, damage to property and internal displacements as they spread to neighbouring provinces in the country. Adopting a qualitative methodology, the case study used a semi-structured questionnaire to interview 50 households who were affected by the disaster. The data were cleaned, coded and descriptively analysed by drawing upon the main themes identified. The study revealed that the floods affected the spiritual and mental health of the survivors as a result of a lack of trust in appointed governmental authorities, feelings of isolation, hopelessness and grief. It became evident from the study that municipalities, with a particular focus on the eThekwini Metropolitan Municipality, need to acknowledge need for spiritual and mental health interventions within the affected communities during and after the disasters. The research contributes to the understanding of human distress and coping mechanisms in the aftermath of flood disasters, by placing the focus on the spiritual and mental health of the survivors.

## Introduction

Globally, it is known that disasters are becoming complex and problematic. Each year individuals and communities are affected by the disasters and the impacts are disruptive to their mental and spiritual wellbeing (Makwana 2019). The United Nations International Strategy for Disaster Reduction (UNDRR 2015) defines disasters as:

[*A*] serious disruption of the functioning of a community or a society, at any scale, due to hazardous events interacting with conditions of exposure, vulnerability, and capacity, leading to one or more of the following: human, material, economic and environmental losses, and impacts. (p. 1)

The above is relevant to the events that unfolded in KwaZulu-Natal during 2022. The devastation within this province prompted a presidential decision to declare this a national state of disaster. Because of the declaration, the municipality of eThekwini received a measure of assistance and resources. The devastation left individuals and communities physically, economically and mentally scarred. Spirituality and religion form an integral part of human existence. The impact on the spiritual and mental health is worsened by the fact that it is not visible in contrast to the economic or financial impacts (Ahmadi 2006; Coppola et al. 2021). It is against this background that the study focused on the spirituality and mental health of the disaster-affected people in KwaZulu-Natal. What started as the normal rainfall season, soon turned streams and rivers into floods, causing devastation (Grab & Nash 2023). The torrential rains destroyed dozens of homes, washed away roads and triggered mudslides. The rainfall caused damage to communication systems, halting education, eradicating water and energy infrastructure and causing businesses and private buildings to collapse. More importantly, lives were lost, where many could not be found, while others were mutilated by the impact of the water. With this magnitude of devastation, the spirituality and mental wellbeing of the community became the point of departure for this study.

### Psychosocial impacts after disaster occurrences

Spirituality, mental health and spiritual wellness contribute to the way flood victims perceive a disaster. Spiritual woundedness has an impact on the mental health of traumatised persons (Van Straten 2019). After an extensive study regarding the various definitions of spirituality, it is necessary to define spirituality within this research to remain focussed within the boundaries of the topic. This study defines spirituality as:

Spirituality is the quest for ultimate meaning, concerned with a sense of connectedness to, and relationship with God, self, others, and the world, towards a feeling of living a fulfilled, hopeful life. (Van Straten)

Therefore, the main concept this study will focus on is meaning making, a relationship with God and a sense of hope, purpose and belonging. A sense of belonging relates to our relationship with self, others and the transcendent, whereas beliefs and values provide a deep sense of purpose and meaning. Sipon et al. (2015) found that flood victims believed that God offers hope and solutions during adversity and suffering and created a sense of belonging among the flood victims while strengthening community relationships. Where religion is guided by tradition, rules and culture, and is a service of worship, spirituality is associated with quality and meaning of life (Paul Victor & Treschuk 2020). Spirituality instils a sense of hope and purpose, which forms part of the coping mechanisms of victims (Sipon et al. 2015). Therefore, religion plays an important role in the mental health and healing of flood survivors.

Weather affects our daily lives, especially within extreme conditions such as floods or drought occurring within a short space of time. These events cause trauma because of damage to property, injuries and loss of life. If unattended to, they may even cause posttraumatic stress disorders (PTSD). According to the Emergency Events database (EM-DAT), of the total number of people affected by weather-related disasters globally, 56% were affected by floods during 1995 to 2015 (Guha-Sapir, Hoyois & Below 2015):

South Africa is no exception and experienced 77 major floods between 1980 and 2010, costing the lives of at least 1068 people. Many severe floods have occurred since 2010 with losses of life, livelihoods, and extensive damage to infrastructure. Recovery has taken years and has required ongoing investment; yet, despite all these endeavours, many communities have not been able to get back to where they were before. (Le Maitre et al. 2019:56)

During April 2019, the coastal province of KwaZulu-Natal (KZN), in the east of South Africa, experienced floods that led to 71 deaths, more than 1400 people displaced and an estimated damage of about USD71 million (Maseko & Wycliffe Muia 2019). KwaZulu-Natal experiences frequent flooding, with heavy rainfall within a short period. According to Engelbrecht (in Wroughton 2022), 12 inches of rain fell in approximately 24 h and resulted in a greater number of fatalities than the 1987 Durban floods where 500 people died. In April 2022, KZN was again severely impacted by devastating floods. The floodwaters swept through homesteads where makeshift huts built from wood and mud were swept away during the night while most were asleep, leaving thousands homeless and destitute or deceased. The official death toll announced by the South African government was 448, yet many people remain unaccounted for. Adding to the flood tragedy is the high unemployment rate, increasing inflation, power blackouts, and a fear of a coronavirus disease 2019 (COVID-19) resurgence (Wroughton 2022).

Funds received from civil society organisations to assist the affected communities were apparently not utilised for this purpose (Ndenze 2022). The Daily News (Phungula 2022) reported that President C. Ramaphosa and the Finance Minister, E. Godongwana, made R1 billion available to rebuild homes and infrastructure damaged by the KZN floods but was later announced to no longer be available for use in this cause. The KZN Cooperative Governance and Traditional Affairs Department appealed to South African citizens to donate funds in lieu of flood relief efforts. However, scepticism exists if these funds would reach the flood victims (KwaZulu-Natal Province 2022). Many destitute victims decided to return to their communities and try to rebuild on their own.

### Spirituality and flood disaster

The trauma caused by being exposed to flooding in KZN increased the already present physical and mental hardships survivors had to cope with because of economic dysfunction and increased their struggle to cope, recover and adapt to the aftermath of the disaster (Reeves 2016). The disaster affected all groups of people, regardless of being religious or not, wealthy, or poor, young or old. Yi et al. (2010) stated that disasters are the great equalisers within any society. This, however, does not take into cognisance the different vulnerabilities of the people within the same community, which clearly manifested in KZN during the floods in 2022. The communities that were more affected and failed to cope with the impact were housed in shelters around the province. Their coping mechanisms are very low, hence they had to turn to the government and other stakeholders such as non-governmental organisations (NGOs), faith-based organisations and the private sector, for relief aid.

### Decisions

A person finds meaning in his understanding of the world (Drenthen 2013). This can be done when one finds a life purpose (Van Lith 2014:21), a sense of belonging (Lambert, Stillman, & Fincham 2013) and finding meaning in experiences (Machell et al. 2015). People need to attach meaning to their experiences and relationships (Park 2013). The loss of meaning may have many causes, of which trauma and stress are among the culprits. Meaning making could influence a person’s well-being (Sales, Merril & Fivush 2013). A basic human need is the existential reality of intimacy. Unconditional acceptance within the realm of human relationship is threatened when official first responders (OFRs), for example, show vulnerability in the workplace, when they struggle to cope with stressful or traumatic incidences and when they suffer from mental illness in the aftermath of a disaster. Spiritual transformation includes an awareness and acceptance of one’s dependence on God (McMinn 2012). The co-researchers found it very troublesome that they had to cope alone, with no support from the government whom they trusted to assist. However, they valued their relationship with God and others, who they thought seemed to be understanding of their plight. According to Pargament (2007), people who believe that they can rely on God and that he is working along with them to solve their problems are more prone to solving stress-related problems and, in the process, leads to the relief of stressful effects (see also Krause & Hayward 2022). The aim of the article is to assess the mental and spiritual health of KZN flood disaster survivors.

## Methodology

The study investigated the spiritual and mental health of flood victims in the Maphumulo and Kranskop area, KwaZulu-Natal province of South Africa during the months of May 2022 and June 2022. A systematic research design that integrated different components of the study in a coherent and logical manner to address the research problem and questions were used (Leedy & Ormrod 2015). A purposive, mixed method qualitative method was followed. Fifty questionnaires were distributed by hand to flood-stricken households, of which 41 agreed to participate. The questionnaires were in English and isiZulu and the completed questionnaires were collected days after receipt. The participants completed the questionnaire measuring their spiritual and mental well-being. The questionnaire consisted of short questions with answers ‘yes’ or ‘no’, or ‘agree’ or ‘disagree’, consisting of 10 negative coping items and 8 positive coping items. The different questions measured inter- and intra-relationship discontent, spiritual connection, a punishing God reappraisal, reappraisal of God’s power, a hope-experience and a sense of purpose. One open-ended question was asked to determine how they perceived the impact of the flood disaster. Close-ended answers were analysed descriptively, and open-ended questions were summarised according to themes.

### Ethical considerations

Permission to conduct the study was obtained by the Ethics Committee of the University of the Free State (GHREC) (No. UFS-HSD2022/0965/22). Participant interviews were conducted accordingly. All participants were older than 18 years. In order to protect the anonymity of the participants that took part in the study, no participant identifiers have been provided.

## Results and discussion

Table 1 summarises the demographic profiles of the participants. Most participants were women (80%) and unmarried (56%). The reason for the small population group was that the victims were mostly homeless, and many moved to live with relatives in different areas. Some of the households that remained lived in tents or with neighbours.

**TABLE 1:** Demographic profile of the participants (*N* = 41).

Demographic data	Frequencies	
	*n*	%
**Areas**		
Maphumulo	36	88
Kranskop	5	12
**Gender**		
Male	8	20
Female	33	80
**Relationship**		
Married	18	44
Unmarried	23	56
**Age (years)**		
20–30	3	7
31–40	12	29
41–50	4	10
51–60	8	20
61–70	10	24
> 70	4	10

Table 2 summarises participants’ views on spiritual negative and positive coping mechanisms. The flood survivors mostly consisted of a Christian community and only one person indicated that he did not worship.

**TABLE 2:** Participants’ views on spiritual negative and positive coping mechanisms (*N* = 41).

Variable	The future does not seem hopeful		I feel very sad		I feel angry		God punished me with the Flood		God is angry at me		I feel that nobody can help me with my problems		I do not feel that prayer will help me with my problems		Talking to the people in my community will not help me with my problems	
	*n*	%	*n*	%	*n*	%	*n*	%	*n*	%	*n*	%	*n*	%	*n*	%
Yes	25	61	34	83	29	71	34	83	10	24	18	44	-	-	-	-
Agree	-	-	-	-	-	-	-	-	-	-	-	-	24	59	21	51
No	12	29	3	7	11	27	6	12	30	73	22	54	-	-	-	-
Disagree	-	-	-	-	-	-	-	-	-	-	-	-	16	39	19	46
No reply	4	10	4	10	1	2	1	2	1	2	1	2	1	2	1	2

According to Maslow’s hierarchy of needs theory, it is meaningless to meet top-level needs if the most basic needs are not met (Maslow & Lewis 1987). A prominent need in this theory is physiological and includes food, drink, air, sleep, clothing, warmth and shelter, which are necessary for survival. Physical hazards such as flood disasters have the potential to threaten these needs. The need for safety occupies level two, where people need to experience control, predictability and economical security. The KZN flood disaster had a devastating effect on the victims, affecting their basic and safety needs.

In the aftermath of the disaster, the moral and emotional well-being of the flood victims are rarely considered. The open-ended questions revealed feelings of resentment towards the government for not intervening and providing support as they had promised. However, the study revealed positive attitudes towards community and neighbours’ social support. A study by Gu, Hu and Wang (2022), which focused on the spiritual experiences, and recovery of flood survivors, and the social support received, found that these aspects were not considered as a vital part of the recovery process.

### Assessing spiritual health of flood survivors

The study revealed feelings of hopelessness and a bleak future. One of the reasons for this was because of a sense of abandonment by the government. This was confirmed by the responses such as: ‘My family suffered from humanity, we see it in the eyes, hungry and suffering. No help from the government. We live in a hall’. Many lost their jobs and had to leave their homes for extended periods to find odd jobs. Feelings of bitterness and sadness are prominent. One participant wrote ‘My country has failed dismally. I have no hope’. Feelings of disconnectedness and abandonment were identified in a participant’s response: ‘I saw that I was abandoned, but in heaven I am not abandoned, I don’t work, I don’t have children’.

Fifty-one per cent of the participants felt that talking to others about their problems will not be beneficial, as there was nobody that could help them (44%), while 46% agreed that talking to others in their community had a positive effect. Research indicated that 88% of the participants believed that the cause of the flood disaster was an act of punishment from God, 73% believed that God was not angry at them and 39% felt that prayer would help them with their problems. This is an indication of spiritual woundedness because of the broken relationship with self and a Transcendent. In this case, God. The outcome shows that, although all the participants, excluding one, indicated that they were Christians, they preferred to talk to each other rather than to God in prayer. Feelings of hopelessness may lead to depression, which in turn creates spiritual disconnection (Sorajjakool et al. 2008).

### Assessing mental health

Emotions of sadness and anger, loneliness, fear, loss, grief and shock were some of the emotions relayed in the questionnaire:

‘I was deeply moved to see people losing their families in such a cruel way and being left homeless.’

‘It pains me a lot to see people die so painfully, even the smallest ones.’

‘I was very shocked to see people dying and small children dying like that.’

Koenig (2009) confirms that many people who suffer from mental illness or emotional problems often seek refuge in religion for comfort, hope and meaning, which essentially confirms the importance of spiritual healing within mental health.

### The way forward, suggestions and recommendations

Spirituality, religion and mental health have been largely neglected when tending to the needs of flood survivors. An understanding and acknowledgment of the spiritual needs of the victims of flood disasters should be included in disaster management intervention program. Disaster practitioners should clearly understand the difference between spirituality and religion and how these terms relate to mental health within disaster-related events. The practitioners need to involve the support groups and churches and other faith-based organisations during disasters such as the floods in KZN. Practical interventions by disaster practitioners might include being visible at ground zero or by visiting the flood-stricken areas and shelters in order for the survivors to experience a sense of presence by government.
